# Radiotherapy in head and neck cancer management: United Kingdom National Multidisciplinary Guidelines

**DOI:** 10.1017/S0022215116000463

**Published:** 2016-05

**Authors:** C Nutting

**Affiliations:** Head and Neck Unit, The Royal Marsden NHS Foundation Trust and Institute of Cancer Research, London, UK

## Abstract

This is the official guideline endorsed by the specialty associations involved in the care of head and neck cancer patients in the UK. Radiotherapy is one of the key treatment modalities used in head and neck cancer management. This paper summarises the current role and some of the recent advances in radiotherapy in head and neck cancer management.

Radiotherapy (RT) and surgery are the two most frequently used therapeutic modalities in head and neck cancer. For early-stage tumours in many sites, surgical excision or RT have similar cure rates but have a different side-effect profile. Radiotherapy traditionally offered higher rates of organ preservation and for some cancers where function is important, it is the treatment of choice. For example, RT allows preservation of natural speech and swallowing in carcinomas of the larynx and tongue base.^1^ At other sites (e.g. carcinoma of the oral cavity), surgical excision alone may be curative and be associated with a very satisfactory functional outcome. The choice of treatment modality therefore depends on individual factors including patient preference.

For an advanced squamous cell carcinoma of the head and neck, single modality treatment (either surgery or RT alone) is associated with poor outcomes. For these tumours, the combined use of surgery and post-operative RT or use of combined chemotherapy and radiation schedules frequently offer the highest chance of achieving cure.

In recent years, RT has benefited from advances in cancer imaging, treatment planning computer software and developments in radiation delivery technology. It is now one of the most technology-driven branches of medicine. Typically head and neck cancer patients will have radiation therapy which is based on the state-of-the-art imaging technology including computed tomography, magnetic resonance imaging, positron emission tomography or other imaging techniques. Optimisation of treatment planning is performed on high-speed computer software, which intelligently selects the most appropriate beam directions and shapes. Treatment is delivered by computer-driven linear accelerators with sub-millimetre accuracy allowing radiation to be focused on the tumour bearing tissues and minimising radiation to normal tissue structures.

Intensity-modulated radiotherapy (IMRT) is now an established form of radiation therapy which allows better control of radiation dose delivery in the head and neck. In a randomised trial performed in the UK, IMRT has been shown to reduce radiation-induced xerostomia (the main long-term side effect of the standard RT) from 75 to 39 per cent (*p* = 0.004) at 12 months following treatment.^2^ A similar result has been demonstrated for patients with nasopharyngeal cancer.^3^ Intensity-modulated radiotherapy in now used to treat 70–80 per cent of patients with advanced head and neck cancer, where sparing of salivary glands is required.

Improvements in local tumour control have been demonstrated with accelerated (delivery of radiation over a shorter time period) or hyperfractionated (delivery of a higher dose of radiation by two to three low-dose fractions per day) RT. These treatments have not shown consistent improvements in overall survival, and for that reason have not been adopted widely outside of North America.^4^

Particle therapy such as with protons or stereotactic RT may allow additional advantages to patients with tumours close to particularly radiosensitive organs such as the brain, spinal cord or in children's cancers. Presently, proton therapy is not available in the UK, but the NHS Proton Clinical Reference Panel can approve treatment abroad for adult head and neck cancer patients with base of skull chordoma or chrodrosarcoma, as well as a variety of paediatric cancers. UK proton facilities at The Christie Hospital in Manchester and University College Hospital in London will be treating patients within the next few years and additional indications for head and neck cancer patients may become apparent based on future research.

In a large meta-analysis of 93 trials and over 17 000 patients, concomitant chemotherapy (given during RT) was shown to improve locoregional control rates and was associated with a 6.5 per cent increase in survival (*p* < 0.0001).^5^^,^^6^ The benefits were largely confined to chemotherapy given during RT rather than the adjuvant or neo-adjuvant setting. In addition, combining chemotherapy with radiation improves the rates of organ conservation. Cisplatin chemotherapy schedules are the most effective.

Similarly the concurrent administration of cetuximab, an anti-epidermal growth factor receptor antibody, with RT, was shown to increase overall survival and locoregional control in this setting.^7^^,^^8^ This was the first demonstration of activity of a biologically targeted therapy in cancer treatment.

In the post-operative setting, two randomised controlled trials have demonstrated the use of concomitant cisplatin during radiation to increase tumour control and overall survival in high-risk patients with positive resection margins or extracapsular lymph node spread.^9^^,^^10^

While concomitant chemotherapy has a proven role in improving outcomes for head and neck cancer, the role of neo-adjuvant chemotherapy remains controversial. Two large studies suggested that the use of docetaxel, cisplatin and 5-fluorouracil prior to definitive RT improved survival.^11^^,^^12^ The use of non-standard RT and/or chemoradiation schedules in these trials has led to uncertainty as to the benefits of this approach when a standard chemoradiation is prescribed. Induction chemotherapy is now becoming less frequently used, and its benefit is probably limited to patients who are at high risk of systemic metastatic spread.

There is increasing evidence that human papilloma virus-related oropharyngeal cancer has an excellent outcome with chemoradiotherapy and that less intensive RT schedules may be more appropriate, which is currently being investigated.

## Key points

• Radiotherapy is a key modality in the treatment of head and neck cancer
• Conformal radiotherapy planning and chemoradiation techniques should be available in all treatment centres
• Intensity-modulated radiotherapy has been shown to reduce long-term xerostomia and should be offered to all appropriate patients.
